# Severe hypoglycemia revealing a münchhausen syndrome by proxy : A case report

**DOI:** 10.1192/j.eurpsy.2021.1603

**Published:** 2021-08-13

**Authors:** L. Aribi, E. Mhiri, N. Messedi, F. Charfeddine, D. Gdoura, J. Aloulou

**Affiliations:** 1 Psychiatry (b), Hedi Chaker University hospital, Sfax, Tunisia; 2 Psychiatry (b), Hedi Chaker University hospital, sfax, Tunisia

**Keywords:** children abuse, maltreatment, Munchhausen syndrome

## Abstract

**Introduction:**

Munchausen syndrome by proxy, is a very rare form of abuse, lying on the border between pediatric, psychiatric and legal fields.

**Objectives:**

To describe a case of Munchausen syndrome by proxy in a mother after the discovery of severe hypoglycemia in her 14-month-old child, hospitalized in the CHU Hédi Chaker Sfax pediatric ward.

**Methods:**

This is a presentation of a clinical case and review of the literature via pubmed using the following keywords : “children, abuse, maltreatment, Munchhausen syndrome”.

**Results:**

This is a 23-year-old woman, mother of two daughters, with a psychiatric history, married to a 43-year-old men known to have diabetes on insulin for several years. The patient was admitted to our ward under constraint for aggressiveness towards her one-and-a-half-year-old daughter. Indeed, one month before her hospitalization, her youngest daughter was hospitalized (accompanied by her mother) in the pediatric department at the Hédi Chaker Sfax University Hospital for severe hypoglycemia (0.3g / l). During hospitalization, the girl presented a more severe hypoglycaemia relapses (0.1g / l) and neurological complications. As the mother was indifferent to her daughter’s troubles and was neglectful of her while caring for the other hospitalized children, a mother-child separation was decided and then the blood sugar levels was stabilized. Besides, several other incidents have been happening repeatedly for the two children. In view of the total history, Münchhausen syndrome by proxy has been mentioned.

**Conclusions:**

Munchausen syndrome by proxy is a complex form of child abuse by the mother, representing a major diagnostic and therapeutic challenge for both somaticians and psychiatrists.

